# Revealing the role of oxidation state in interaction between nitro/amino-derived particulate matter and blood proteins

**DOI:** 10.1038/srep25909

**Published:** 2016-05-16

**Authors:** Zhen Liu, Ping Li, Weiwei Bian, Jingkai Yu, Jinhua Zhan

**Affiliations:** 1 Key Laboratory of Colloid and Interface Chemistry, Ministry of Education, Department of Chemistry, Shandong University, Jinan 250100, China; 2 National Key Laboratory of Biochemical Engineering, Institute of Process Engineering, Chinese Academy of Sciences, Beijing 100190, China; 3 University of Chinese Academy of Sciences, Beijing 100049, China; 4 Department of Pharmacy, Weifang Medical University, Weifang 261053, China

## Abstract

Surface oxidation states of ultrafine particulate matter can influence the proinflammatory responses and reactive oxygen species levels in tissue. Surface active species of vehicle-emission soot can serve as electron transfer-mediators in mitochondrion. Revealing the role of surface oxidation state in particles-proteins interaction will promote the understanding on metabolism and toxicity. Here, the surface oxidation state was modeled by nitro/amino ligands on nanoparticles, the interaction with blood proteins were evaluated by capillary electrophoresis quantitatively. The nitro shown larger affinity than amino. On the other hand, the affinity to hemoglobin is 10^3^ times larger than that to BSA. Further, molecular docking indicated the difference of binding intensity were mainly determined by hydrophobic forces and hydrogen bonds. These will deepen the quantitative understanding of protein-nanoparticles interaction from the perspective of surface chemical state.

Epidemiologic research reveal the complexities and uncertainties in identifying the relationship of fine particulate matter (PM_1, 2.5, 10_) with various physiological dysfunction^1^,^2^. For individual particles, the critical question is the multi-parameters on phases, size distribution, chemical composition and surface state^3^,^4^. Ultrafine PM are mainly generated from incomplete combustion of engine, which consisted of a core of elemental carbon and some adsorbed layers of organic hydrocarbons, metals, nitrates, and sulfates^5^. The life of these carbon soot are regional-scale and time-scale^6^,^7^. They can be chemical coupling and transformed to secondary particulate matter in the heterogeneous photochemical reactions by stratospheric radiation-derived active species, such as NO_x_, hydroxyl radical (OH•) or monoatomic oxygen^8^,^9^,^10^. Those heterogeneous reactions change the surface chemical composition of particles, making it get more active surface state^11^,^12^. However, these changes will influence the interaction with biomolecules (protein, lipid and saccharides), to some extent, that is believed the important questions of the transport, distribution, metabolism and potentially toxic effects in tissues.

Recent preclinical studies confirmed that PM-induced oxidative stress is an important cause for the acute inhalation pulmonary injure^13^,^14^. The active molecule on carbon soot can act as a catalyst to generate excess reactive oxygen species (ROS) by the aerobic metabolism and body defense mechanism^15^,^16^,^17^. High ROS levels induce proteins, DNA damage and tissue inflammation^18^,^19^,^20^,^21^. The surface oxidation state of particles may influence their potentially toxic effects in the tissues. In lungs, carbon soot penetrate into alveolar membranes, the surface nitro group would be reduced to amine through six-electron reactions by cytosolic nitroreductases and microsomal cytochrome P450s^22^,^23^. The nitro and amino induce both qualitatively and quantitatively different effects on cytokine/chemokine expression in bronchial epithelial BEAS-2B cells, that may trigger pro-inflammatory responses through different initiation mechanisms^24^,^25^. The oxidation state of surface group could have an influence on the conjugation of protein^3^,^26^. Therefore, it is valuable to evaluate the impact on binding mode and intensity, which will promote the comprehensive understanding on transport and metabolism in blood circulation system.

Although the actual sample can provide more valuable information, considering the challenge on separation and analysis, modeling analysis have been used in particulate pollutants evaluation^27^. Surface oxidation state of actual carbon soot would be more complex. In this study, the amino/nitro groups had been selected as an example to evaluate the impact on interaction with blood proteins from the perspective of molecular interaction. Gold nanoparticles (AuNPs) can be high-quality synthesized with controlled size and shape. They are stable enough in solution, and also can be decorated by different ligands to get desired surface properties. These advantages make it useful for biosensor or biomedicine. It is also an ideal material for the study of surface effect of nanoparticles with biomolecules^28^. AuNPs also have been selected as model to research the influence of surface charge density on uptake of nanoparticles by cells^29^. Many analytical methods could been applied to monitor the particles-proteins interaction^30^,^31^,^32^. Capillary electrophoresis (CE) has been illustrated as a powerful tool for monitoring the dynamic association/dissociation of protein-ligand binding and complex formation^33^. Affinity capillary electrophoresis (ACE) technique integrate the advantages of separation and analysis, makes it highly suitable for qualitative and quantitative measurement of affinity in physiological environment, particularly, for the unlabeled protein assessment *in vitro*^34^. Here, AuNPs was synthesized and modified by 4-aminothiolphenol (ATP) and 4-nitrobenzenethiol (NBT) to model the surface oxidation state of carbon soot. Then, the interaction of particles with serum albumin (BSA) and bovine hemoglobin (BHb) were investigated by CZE and ACE quantitatively, giving the dissociation constants (K_D_) and cooperativeness coefficients (n) according to the Hill equation. Further, the binding sites and force were evaluated by molecular docking. Results demonstrate the surface oxidation state can affect the intensity and mode of protein conjugation.

## Results

### Stability in solution

Firstly, the stability of thiophenol-capped AuNPs was investigated by UV-vis absorption spectra in the buffer solution. Five mole rate samples were prepared by mixing AuNPs and ligands stock solution in the range of 10:1, 10^2^:1, 10^3^:1, 10^4^:1 and 10^5^:1. The absorption spectra for each sample is shown in Supplementary Fig. 1. Citrate AuNPs are stable in solution and have a characteristic plasmon band at 520 nm. The plasmon spectrum is sensitive to the nanoparticle surface charger, as the concentration of thiophenol increased, nanoparticles surface potential switched up to relatively positive (refer to the zeta potential date in supporting information). The aggregation will lead to dipole coupling of the plasmon between neighboring particles, the color of the solution changes from wine red to blue (Supplementary Fig. 1a)^35^, the band of AuNPs shifted to a new longer wavelengths at 750 nm for ATP. No aggregation was observed as the concentration increased in NBT-AuNPs system, the nitro group could play the citrate role to stabilize the AuNPs (Supplementary Fig. 1b).

### SERS of ATP/NBT capping AuNPs

The plasmonic coupling between the nanoparticles could result in strong electromagnetic enhancement for the ligand molecule. For more information about the surface chemical component on AuNPs, SERS spectra was employed to confirm the ATP and NBT ligands. As shown in Supplementary Fig. 2, the strongest band at approximate 1077 cm^−1^ is attributed to the stretching vibration of C-S, and the band at approximate 1580 cm^−1^ is C-C stretching vibration of benzene rings. In detail, the Raman spectra peak at 390 cm^−1^ were attributed to the a_1_ model of the ATP, the peak at 1171 and 1489 cm^−1^ belong to the b_2_ model (Supplementary Fig. 2a), the peak at 1344 and 1636 cm^−1^ are assigned to the a_1_ model of NBT (Supplementary Fig. 2b)^36^,^37^. Raman intensity increases with the increasing concentration of ligand, especially, the great Raman signal was observed at the critical concentration ratio of 10^3^:1. It is concluded that the thiophenol molecules bonded to the Au surfaces nearly vertically via formation of the Au-S bond. The maximum occupation number of thiophenol molecule on one gold nanoparticles could be calculated as 1380. At the concentration ratio of 10^2^:1, the surface of the nanoparticles was not wholly covered with thiophenol layer, the residual 2-hydroxypropane-1, 2, 3-tricarboxylate could protect the colloidal gold nanoparticles from aggregation in buffer solution. Therefore, the thiophenol capped nanoparticles will be stable over a wide range of ionic strength and pH for following CE analysis.

### BSA-ATP/NBT system

**Figure 1:**
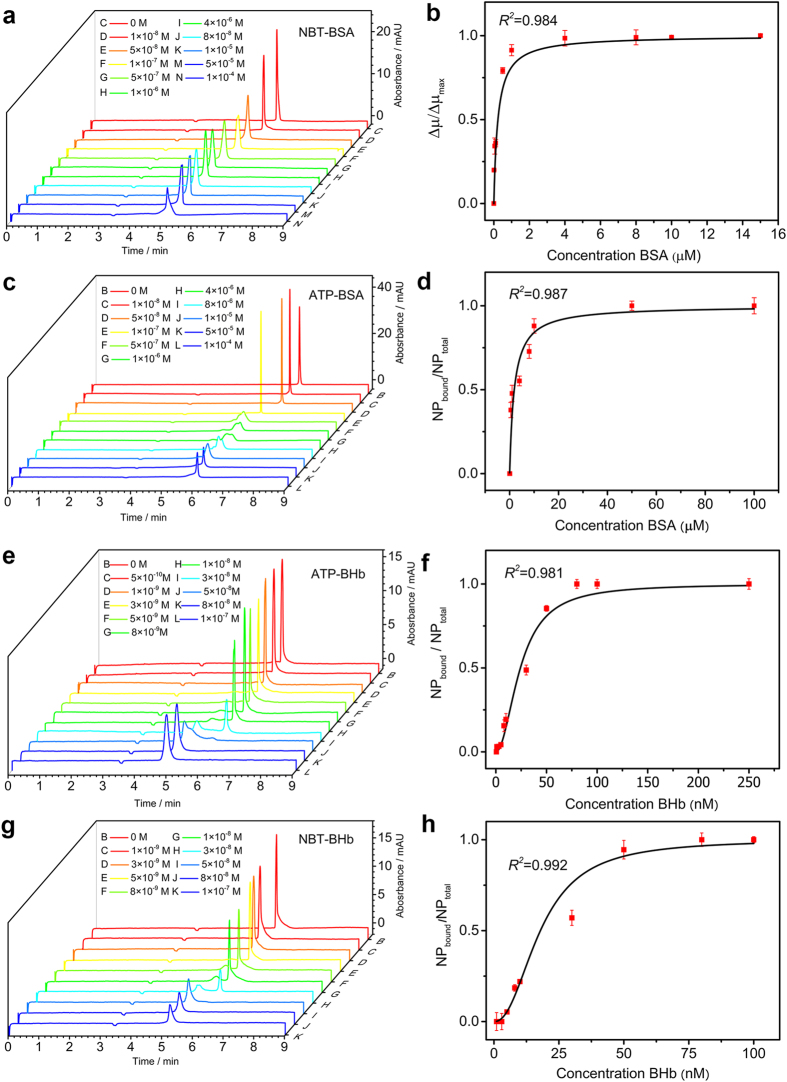
(**a**) ACE analysis of the interaction for BSA (the concentration in running buffer: 1.0×10^−8^ M to 1.0×10^−4^ M) and NBT (1.0×10^−9^ M). CZE analysis of the interaction for (**c**) BSA (1.0×10^−8^ M to 1.0 ×10^−4^ M) and ATP (1.0×10^−9^ M), (**e**) BHb (5.0×10^−10^ M to 1.0×10^−7^ M) and ATP (1.0×10^−9^ M), (**g**) BHb (1.0×10^−9^ M to 1.0×10^−7^ M) and NBT (1.0×10^−9^ M). The corresponding Hill equation fitting curve for (**b**) NBT-BSA, (**d**) ATP-BSA, (**f**) ATP-BHb, (**h**) NBT-BHb, the red error bars correspond to the triple standard deviation methods for three independent replicates. The slight negative peaks at around 3.6 min were belong to the EOF marker ethanol.

In CE process, no distinct complex peak was observed in interaction of NBT capping AuNPs with BSA, confirmed that their model was fast dissociation system. The interaction was investigated by ACE in phosphate buffer (20 mM, pH 7.5) with BSA concentration ranging from 10.0 nM to 10.0 μM. The curve fitting results are shown in Fig. 1a. The first slight negative peaks were EOF maker and the migration peaks after the EOF marker were the AuNPs peaks. The AuNPs migrated faster in capillary as the BSA concentration increased gradually, then the retention time reached the minimum at 1.0 μM (BSA). BSA were negatively charged under conventional neutral physiological conditions, the dynamic bound could speed up the migration of AuNPs. The peak broadening was also observed just the protein was injected, that indicating the absorption of BSA on capillary wall. Figure 1c show the electropherograms of the interaction between ATP-AuNPs with protein, from that one can observe that a two distributions broad peak appear at the BSA concentration increased to 0.5 μM, the prior slight peak was identified as the BSA-particles complex, the second was particles peak, and that the complex peak increased with the BSA concentration, while the AuNP peak decreased gradually and disappeared at 10 μM finally. Therefore, interactions of ATP-AuNPs and BSA can be attributed to the quasi-slow dissociation model, the K_D_ and n of particles-protein complex can be obtain according to the fitting of Hill equation (Fig. 1b,d). The affinity decreased with the surface oxidation status of particles, the NBT have a K_D_ value 0.138 μM, the ATP have a K_D_ value 1.57 μM, the binding cooperativeness n value were 0.73 and 0.78 for the two types of AuNPs.

### BHb-ATP/NBT system

Interactions between BHb and the model particles were typical slow dissociation systems. The association endow the bound AuNPs with faster migration than that free one. The protein-particles complex can be successfully separated and the distinct bound AuNPs peaks were observed. After incubation, the solution was injected and resolved in the 20 mM pH 8.0 borate buffer solution. The broaden peak after the EOF maker was attributed to the complex, this peak increased with the protein concentration. For ATP-AuNPs, the bound AuNPs appeared at the BHb concentration 0.01 μM, and the peak capacity reached the maximum at 0.08 μM (Fig. 1e). For NBT-AuNPs, the bound peak were first detected at the protein concentration 50.0 nM (Fig. 1g). The K_D_ and n of AuNPs-protein complex were calculated using the Hill equation in Fig. 1f,h. The NBT capping AuNPs get a K_D_ value 14.81 nM, the ATP capping AuNPs have a K_D_ value 24.23 nM, the binding cooperativeness n value were 2.08 and 1.98, respectively.

### Binding sites in BSA and BHb

**Figure 2:**
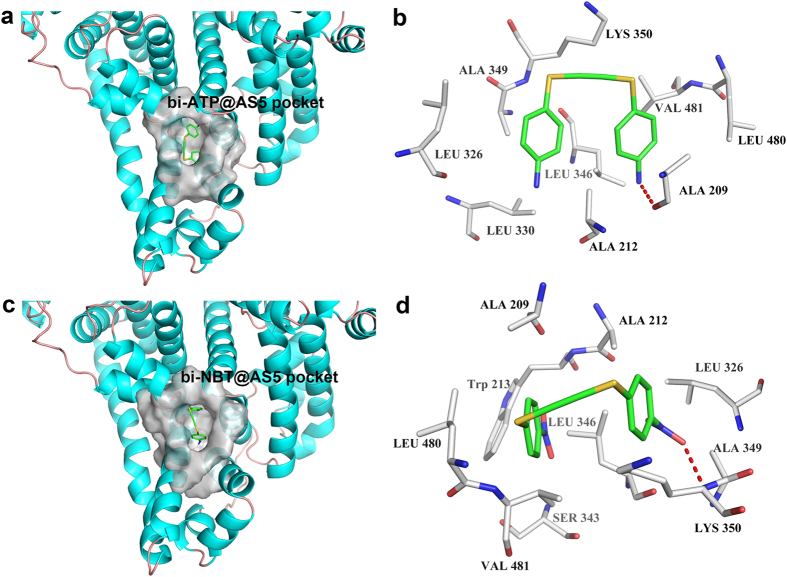
Crystal structure of bi-ligand with BSA. Surface complementation between BSA with (**a**) bi-ATP and (**c**) bi-NBT at AS5 pocket region (gray) are depicted in carbon representation, the ligands is shown in sticks view. Stereo views of the interface details of (**b**) bi-ATP and (**d**) bi-NBT, the contact residues are labeled by type and number, key hydrogen bonds are shown by red dash line, the molecule is colored by atom type, (C, hoary for residues, green for ligands; N, blue; O red). The hydrogen bonds are shown by red color dotted line.

**Figure 3:**
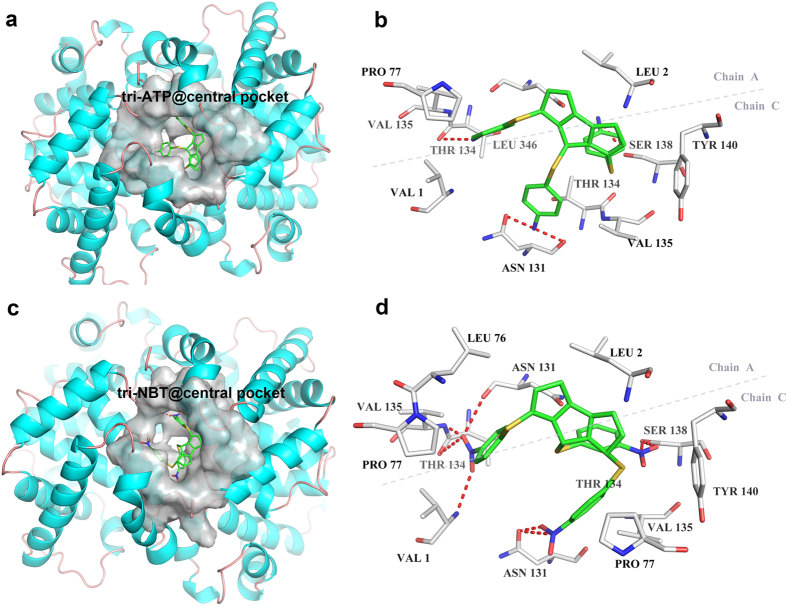
The binding pockets of BHb with the (**a**) tri-ATP and (**c**) tri-NBT ligands at the central cavity is depicted in surface representation (gray), the ligands is shown in stick representation. Stereo views of the interface details of (**b**) tri-ATP and (**d**) tri-NBT, the lable and colour is similar to Fig. 2.

The AS5 binding pocket of BSA is located at the interface between subdomains IIA and IIB. The bi-ATP molecule rotates to the vertical position with twin phenyl planar angle 92.3° and then inserted the cavity (Fig. 2a). One amino of bi-ATP creates single hydrogen bond with the oxygen atom of carboxyl group of Ala 209 in IIA/helix2, and the rings make hydrophobic interactions with the surrounding leucine (Leu303,Leu326, Leu480), alanine (Ala212, Ala349,), Lys350 and Val481 residues by CH-π bonds (Fig. 2b). The bi-NBT molecule binds into the pocket with twin phenyl planar angle 89.9°, one nitro group of ligand creates single hydrogen bond with the main-chain amides moiety of Lys350 in IIB/helix7 (Fig. 2c), the other interacts via π-π bond with the side-chain indole ring of Trp213 in IIA/helix2. The twin rings are stabilized by hydrophobic interactions with surrounding Ala209, Ala212, Ala329, Leu326, Leu346, Leu480, Val481 and Ser343 residues (Fig. 2d). In the BHb system, the structure of tri-ATP molecule was optimized with two dihedral angle of tri-phenyl planar 56.6° and 147.3°, it bound into the central sulcus constituted by α_1_ and α_2_ subunits (Fig. 3a). One amino make two hydrogen bonds with carboxyl group of Asn 131, another bond with hydroxyl group of Ser 138 in α_2_ subunit helix 9, the last one bond with main chain carboxyl group of Thr 134 in α1 subunit helix 9. The tri-ATP ligands interactes with phenyl of Tyr 140 and pyrryl of Pro 77 by π-π bonds, make hydrophobic interactions with the leu2, leu346, Val1 and Thr134 (Fig. 3b). In the BHb-NBT, the dihedral angle of tri-phenyl planar change to 50.6° and 160.3, the first nitro group of ligands bind with carboxyl group of Thr 134, amides moiety of Val135, carboxyl group of Asn131 in α_1_ subunit and N-terminal of Val1 in α_2_ subunit by four hydrogen bonds (Fig. 3c). The N and O of second nitro and third nitro group bound by two hydrogen bonds with the hydroxyl group of Ser 138 and the carboxyl group of Asn 131 in α_2_ subunit, respectively. The phenyl rings is stabilized by π-π bonds with Tyr 140 and Pro 77, and make hydrophobic interactions with the Leu 2, Leu 76 and Val 135 (Fig. 3d).

## Discussion

**Figure 4:**
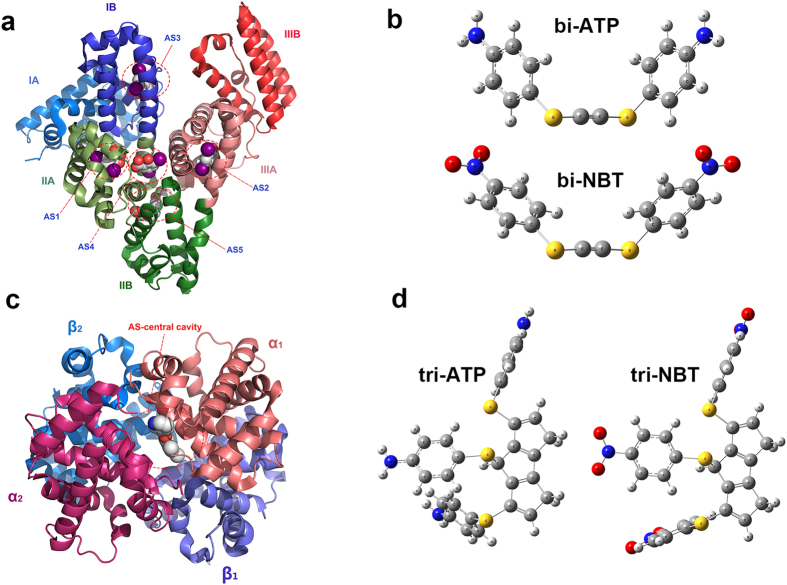
Domain structure of (**a**) BSA, (**c**) BHb and the proposed location of binding sites. The domains I, II and III of BSA are colored blue; green and red, the subdomains A are lighter than B, the proposed binging sites are coded as AS #. The α1, α2, β1 and β2 subunits of BHb are colored orange, magenta, violet and blue, the binging sites are located at the central cavity between α1 and α2 subunits. The cartoon diagram of BSA (PDB ID: 4JK4) and BHb (PDB ID: 3R5I) are generated by PyMOL^TM^ v1.7 sofeware. The optimized 3D stucture of (**b**) bi-ATP, bi-NBT and (**d**) tri-ATP, tri-NBT are shown in sphere representation, the molecule is colored by atom type (C, grey; N, blue; O, red; S, yellow; H, hoary).

The conjugation of proteins mainly depends on the surface properties of nanoparticles at the bio-nano interface. It is believed that different affinity of proteins are mainly caused by the binding ability of two types of ligands. Molecular docking study have been used for evaluating the interaction force and dissociation constant of the two ligands. The larger size of both AuNPs and protein makes the ligands to only interact with the surface active site of proteins^3^. AS5 binding site, located at the interface between subdomains IIA and IIB of BSA (Fig. 4a), had been proved as the pocket for small molecule, such as fatty acids^38^,^39^. In this study, the AS5 site was selected as the potential binding sites for docking. Likewise, a surface pocket at the central cavity of the α_1_ α_2_ subunits is selected as potential binding sites for BHb (Fig. 4c)^40^. According to their corresponding pocket size, 4-aminothiophenol/4-nitrobenzenethiol were reconstructed into bi-amino/nitro ligands by ethynyl and tri-amino/nitro ligands by ternary heterocycle (Fig. 4b,d), respectively.

Size and surface properties are important determinant factors for affinity and cooperativity of particles-protein interaction. Those forces determine the nano-bio interaction in water solution involves van der Waals, electrostatic attraction, hydrogen bonding, solvation forces. In this study, the binding of particles-protein was carried out at the certain pH, ionic strength and solvent properties. The physical size was 15 nm from TEM date (Supplementary Fig. 3). The hydrodynamic diameter of nitro-AuNPs and amino-AuNPs were measured by DLS methods in PBS buffer solution, the value were 35.08 nm (nitro-AuNPs) and 29.25 nm (amino-AuNPs), the zeta potential of two particles were −62.8 mV and −58.9 mV, respectively (Supplementary Fig. 4), this attributed to the partially substitution of thiophenol on particles surface. These results indicate that the size of two particles are similar, the surface charge are both negatively. On the other hand, the results shown high affinity interactions between model particles and protein, the K_D_ values for amino mode (ATP-BSA) was 1.57 μM and for the nitro mode (NBT-BSA) was 0.138 μM. The affinity decreases as the ligand nitro reduced to amino, the oxidation state was 10 times larger than the reduction one, and the corresponding scoring results from docking also proved the tendency (Supplementary Table 1). The cooperativeness coefficient n were calculated as 0.73 and 0.78, indicated the negative cooperativity effects for both of the model particles. BSA was composed of three homologous domains (I–III) and each domain in turn is the product of two sub-domains (A, B)^41^. The primary binding site is situated in the hydrophobic core of sub domain IIA and IIIA, which bound mostly with neutral heterocyclic compounds and aromatic compounds by electrostatic attraction, hydrogen bonding, or van der Waals interactions^42^. According to the literature reported, the isoelectric point of BSA and BHb are 4.9 and 6.8 at 25 °C^43^. In pH 7.5 buffer solution, the two proteins can also get the negatively surface charge. Therefore, the electrostatic repulsion between particles and proteins will oppose their binding. On the other hand, previous studies suggested that the hydrophobic forces and hydrogen bonds played major roles in the binging between nitrobenzene compounds and serum albumin^44^,^45^,^46^. As the docking results indicated, the oxygen atoms of -NO_2_ group have more electronegativity than the nitrogen atom of -NH_2_ which can form more hydrogen bonds with the residue in binding sites. Likewise, the electron-withdrawing power of -NO_2_ group make the aromatic ring get relative positive electrostatic potential^47^. It is believed that the interaction between nitro/amino-AuNPs with ptoteins are mainly influence by hydrogen bond and hydrophobic interactions. In BHb system, the distinct peaks of bound particles were detected in electrophoresis, and the calculation results shown high binding propensities between protein with both of the two particles and had average K_D_ values 10^3^ times lower than that of BSA system. The cooperativeness coefficient were also obtained as the value of 2.08 and 1.98, revealed the positive cooperatives effects. According to the binding pocket size, the central cavity of BHb can bind with three ligands at the same time, which is larger than the AS5 of BSA. The docking results also indicated that any one of ligands can make more hydrogen bonds with residues in BHb than that in BSA (Fig. 3). These may be responsible for the highly strong affinity between BHb and model particles.

The effect of surface oxidation state on particles-protein conjugation intensity and model were quantitatively evaluated by CZE and ACE methods. The oxidation state determined the conjugation intensity and model, towards BSA, the nitro-particles shown larger affinity than amino-particles, the accumulation would resist the transportation and make metabolism difficult in the vascular system. For BHb, both of the two particles shown very high binding tendency, indicated the positive cooperatives effects. The binding with hemoglobin may influence the oxygen-carrying capacity of blood. These will promote the understanding for PM-related cardiovascular effects from the perspective of chemical molecular interaction.

## Methods

### Characterization of Model Particles

The morphology of AuNPs were obtained using a JEOL 2100 transmission electron microscope with an acceleration voltage of 200 kV. The UV-vis absorption spectra were collected on Shimadzu UV-2550 spectrophotometer over a range of 350–850 nm. The Raman spectra excited at 785 nm were collected using an Ocean Optics QE65000 Raman Spectrometer with the maximum excitation of 455 mW, integration time was 1 s. Inductively coupled plasmas atomic emission spectroscopy (ICPAES) tests were performed on a Perkin-Elmer Optima 2000 DV optical emission spectrometer to measure the concentration of Au element. With the size measured under TEM and the element content obtained from ICP-AES, molar concentration of the AuNPs stock solutions were obtained. Zeta potential and hydrodynamic size were measured with a Zetasizer nano system (Malvern, UK) at 25 °C.

### Design of Model Particles

Gold colloid solutions (15 nm AuNPs) were synthesized by the Turkevich-Frens method^48^,^49^. The thiophenol capping AuNPs were prepared by following process. AuNPs solution was centrifugated at 10000 rpm for 10 min, the precipitate was redissolved in pH 7.5 10 mM PBS buffer solution. An ethanol solution of thiophenol (10 μL, 10 μM) was added to the solution of AuNPs (1.0 mL, 10 nM) to give a final concentration of total thiophenol of 0.1 μM. The mole ratio of thiophenol and AuNPs in the mixture is 100:1. Then, the mixture was incubated at 25 °C for 12h. Finally, BSA and BHb at different concentrations were incubated with thiophenol capping AuNPs overnight at 37 °C.

### Capillary Electrophoresis

The prepared protein-conjugated nanoparticles were analyzed on open column capillary electrophoresis system. (TriSep™-2100 high voltage source and EASYSEP Vis-UV detector, Unimicro Technologies, USA). The 75 μm i.d. fused-silica capillary (60 cm total length, 50 cm effective length, Polymicro Technologies) was sequentially rinsed with 0.1 M NaOH solution for 1 h, deionized water for 1 h, and the running buffer for 30 min before analysis. Electrophoresis was conducted with a constant voltage of 20 kV for 9 min at 25 °C using ethanol as the EOF marker. All solutions were degassed under sonication just before use. Capillary zone electrophoresis was performed in 20 mM borate buffer at pH 7.5, protein-conjugated particles were injected at 20 kV for 5 s before separation. In affinity capillary electrophoresis, the running buffer was 20 mM phosphate buffer at pH 7.5 with the protein in concentrations ranging from 10.0 nM to 100 μM for BSA, a solution of 10.0 nM NBT-AuNPs were inject at 20 kV for 5 s.

### Molecular docking and scoring

Molecular docking was performed to obtain the binding affinity and the binding sites were analyzed. Molecular docking calculations were performed with the AutoDock 4.0 package^50^,^51^. The Gasteiger partial charges were assigned to the ligand and the receptor using AutoDock tools 1.52. AutoDock generates different ligand conformers using a Lamarkian genetic algorithm (LGA). All single bonds of the ligand were treated as rotatable during the docking calculation, and altogether 12 flexible torsions were defined. The structure of bi-amino/nitro ligands and tri-amino/nitro ligands was obtained by ChemAxon software, optimized by OpenBabel 2.3.0 using MMFF94 force field model and then used for the docking studies^52^. The center of the grid box was set to the center of complex of ligands and protein, the box size was set to 60 × 60 × 60 with grid spacing 0.375 Å in each dimension, which is large enough for the free rotation of the ligand. Each docking calculation generated 100 structures. The AutoDock scoring function includes terms accounting for short-range van der Waals, electrostatic interactions, and dissociation constant and hydrogen bonding were selected according to the size of clusters RMSD and the estimated free energy of binding (FEB). The PDB structure of BSA (PBD ID: 4JK4, 4OR0) was used for searching the potential binding sites and docking. PDB structure of bovine hemoglobin (PBD ID: 3CIU) was used for docking assessment, human hemoglobin structure (PDB ID: 3R5I) was served as a template for the binding sites.

## Additional Information

**How to cite this article**: Liu, Z. *et al*. Revealing the role of oxidation state in interaction between nitro/amino-derived particulate matter and blood proteins. *Sci. Rep.* **6**, 25909; doi: 10.1038/srep25909 (2016).

## Supplementary Material

Supplementary Information
