# Supplementary material for: Revealing the role of oxidation state in interaction between nitro/amino-derived particulate matter and blood proteins
Source: Sci Rep. 2016 May 16;6:25909. doi: 10.1038/srep25909 (PMC4867627; doi:10.1038/srep25909)
Supplement: Supplementary Information [file srep25909-s1.pdf]

# Revealing the role of oxidation state in Interaction between nitro/amino-derived particulate matter and blood proteins

Zhen Liu[1], Ping Li[2], Weiwei Bian[3], Jingkai Yu[2] & Jinhua Zhan[1*]

**Supporting Note 1**

**Interaction Modes.** The CE measurement of the binding affinity in particles-proteins system following the reported methods, the dissociation equilibrium constant ($K_D$) of the complex can be calculated from the Hill equation [1-3].

$$\theta = [\text{protein}]^n/(K_D{}^n + [\text{protein}]^n) \qquad (1)$$

In equation (1), $\theta$ *is* the protein-bound fraction, $K_D$ is microscopic dissociation constant, describing the affinity of each binding site to the protein, and n represents synergistic effect for the multivalent binding interaction on the particles surface. In slow dissociation system, the relative stable particles-protein complex can be separation and identified from the unbind ones, $\theta$ *value* is equal to the fraction of the protein bound particles in total particles. In fast dissociated system, $\theta$ is calculated from the apparent electrophoretic mobility ($\mu_{app}$) of particles by ACE following the equation 2 [4,5]

$$\mu_{app} = f_1 \cdot \mu_{max} + f_2 \cdot \mu_{free}$$

$$\theta = NP_{bound}/NP_{total} = f_1 = (\mu - \mu_{free})/(\mu_{max} - \mu_{free}) \qquad (2)$$

$f_1$ and $f_2$ are the fractions of protein-bound particles and free particles, $\mu_{free}$ and $\mu_{max}$ are the electrophoretic mobility values when there is no protein and saturated protein.

**Supporting Note 2**

**Materials.** 4-aminothiophenol (97%), 4-nitrobenzenethiol (90%), bovine serum albumin lyophilized powder (66,000 Da) and bovine hemoglobin (64,500 Da) were purchased from Sigma Aldrich. $HAuCl_4 \bullet 4H_2O$ and trisodium citrate were obtained from Sinopharm Chemical Reagent (China). HPLC grade ethanol were obtained from Dikma Technologies, Milli-Q water (18.2 MΩ) was used in all experiments.

**Supplementary Table 1 |** Fitting parameters of protein-particles model

| | BSA | | | BHb | | |
|---|---|---|---|---|---|---|
| | $K_D$ | n | $R^2$ | $K_D$ | n | $R^2$ |
| **ATP-AuNP (Bi-ATP, Tri-ATP)** | $1.57\times10^{-6}\pm3.57\times10^{-7}$ ($4.68\times10^{-4}$) | 0.73±0.10 | 0.9576 | $2.42\times10^{-8}\pm2.2\times10^{-9}$ ($5.43\times10^{-5}$) | 1.98±0.25 | 0.9812 |
| **NTP-AuNP (Bi-NBT, Tri-NBT)** | $1.38\times10^{-7}\pm3.10\times10^{-9}$ ($4.964.68\times10^{-5}$) | 0.78±0.12 | 0.9804 | $1.48\times10^{-8}\pm9.4\times10^{-10}$ ($4.22\times10^{-6}$) | 2.08±0.21 | 0.9728 |

**$K_D$ -dissociation equilibrium constant, n-synergistic effect constant**

**$K_D$ in brackets is the scoring data by docking.**

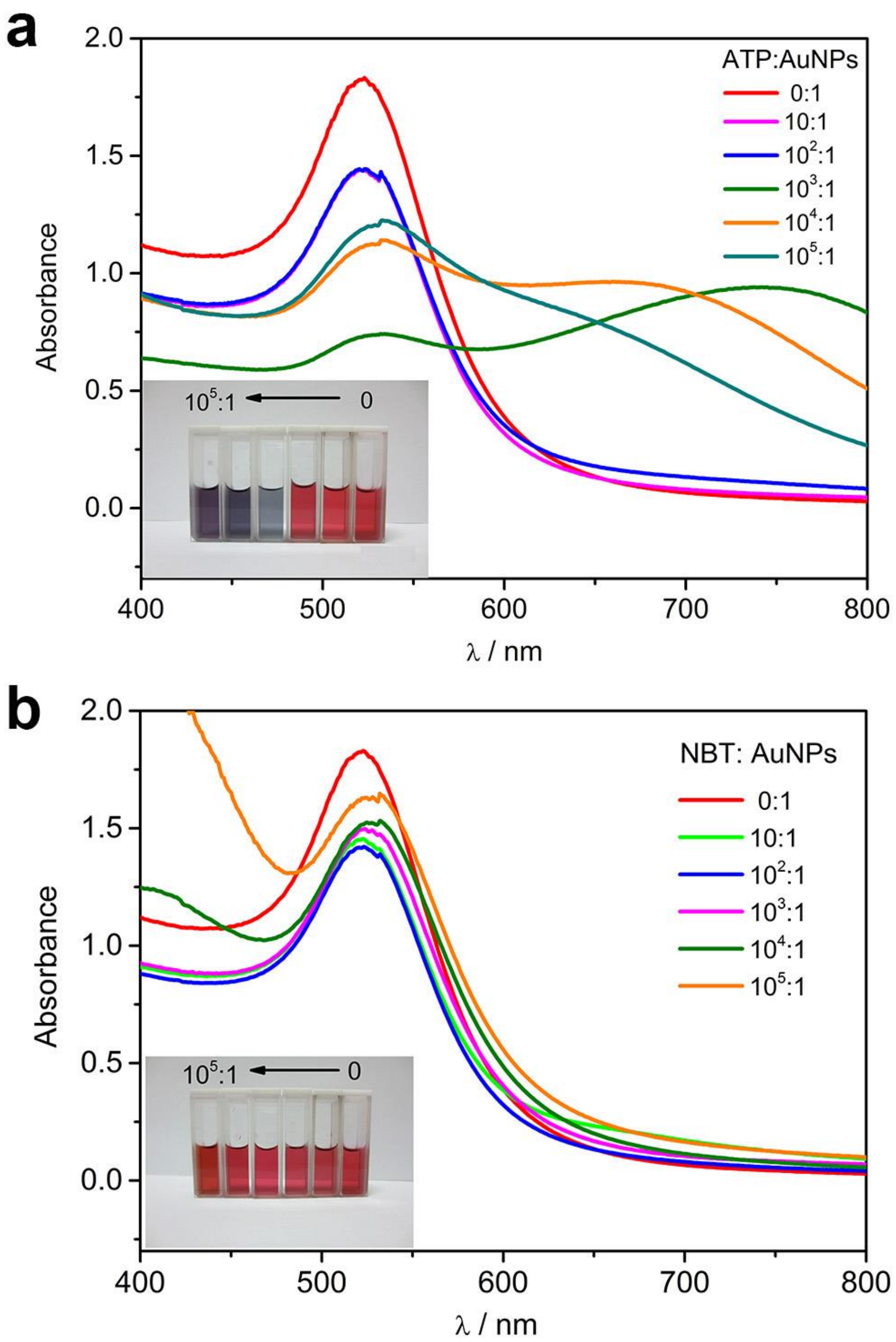


**Supplementary Figure 1 |** UV-vis absorption spectra of AuNPs (10 nM) with different mole ratio ($10^5$:1, $10^4$:1, $10^3$:1, $10^2$:1, 10:1) of ligand stock solution for 4-Aminothiophenol (a) and 4-Nitrobenzenethiol (b).

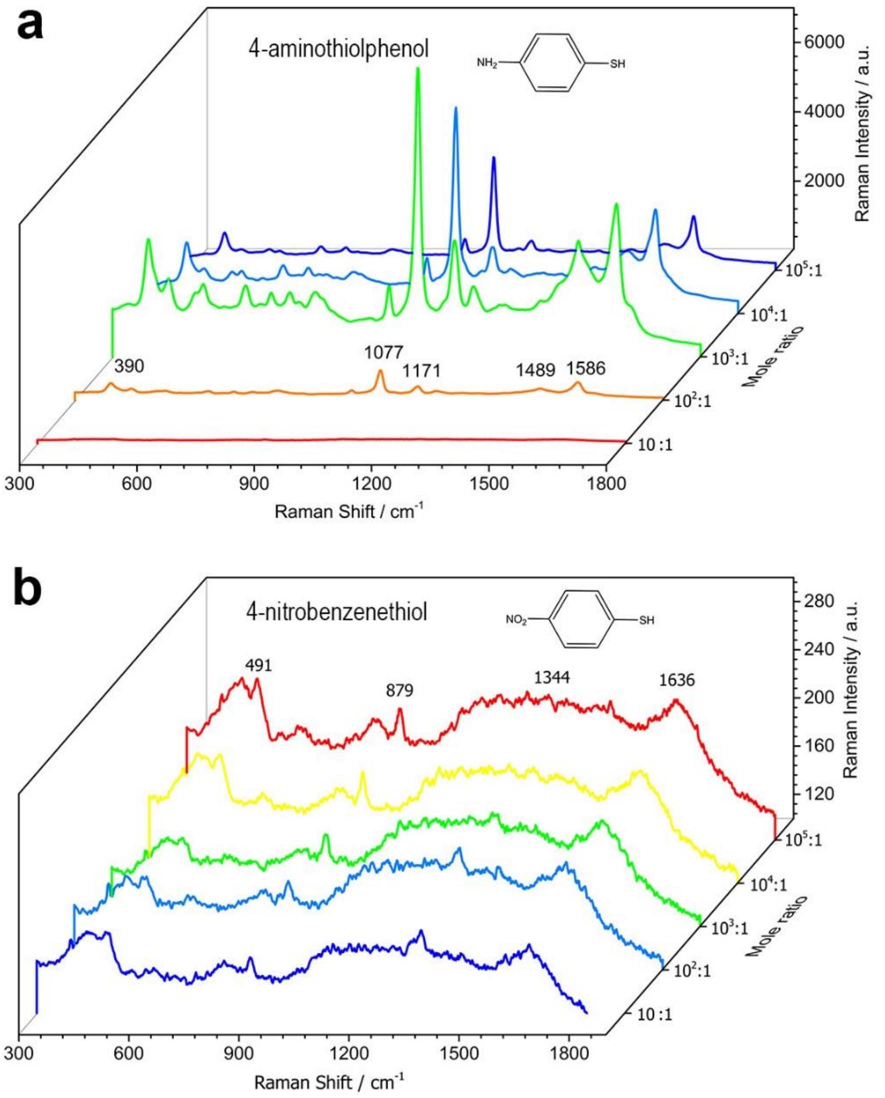


**Supplementary Figure 2 |** SERS spectrum of (a) 4-aminothiophenol and (b) 4-nitrobenzenethiol capping AuNPs with different mole ratio ( the concentration gradient is similar to Supplementary Figure 1). Excited laser: 785 nm, 455 mW.

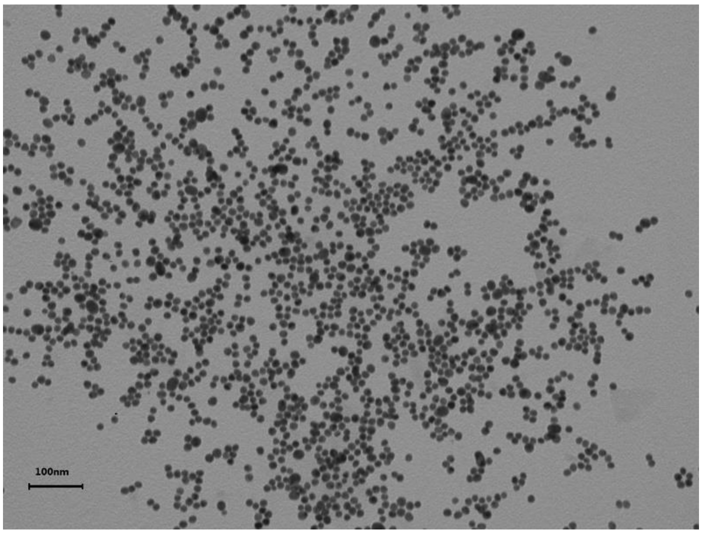


**Supplementary Figure 3 |** TEM of the prepared citrate-stabilized AuNPs.

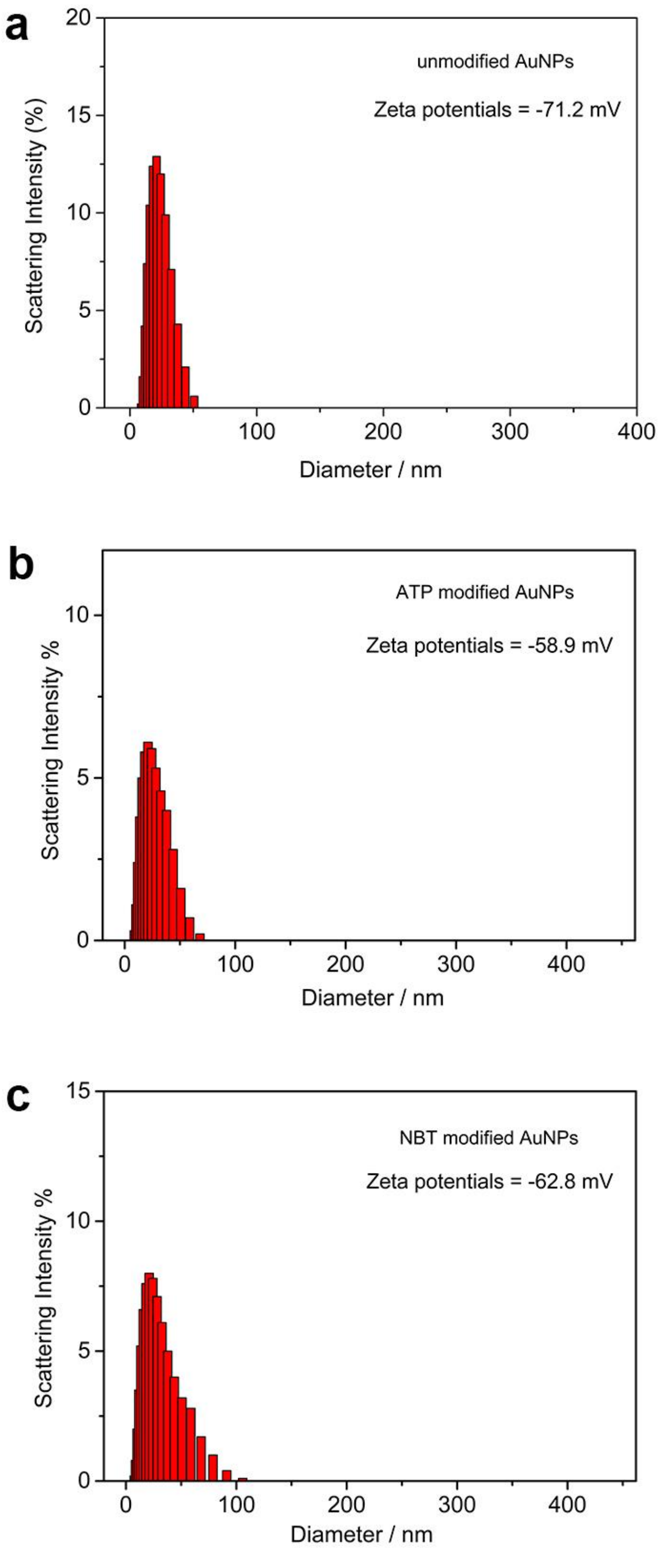


**Supplementary Figure 4 |** Zeta potentials and size distributions of gold nanoparticles measured by DLS in pH 7.5 50 mM PBS buffer solution: (a) citrate-stabilized AuNPs, (b) ATP modified AuNPs, (c) NBT modified AuNPs.

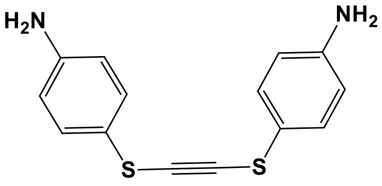

**Bi_amino**

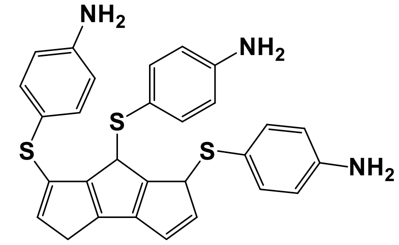

**Tri_amino**

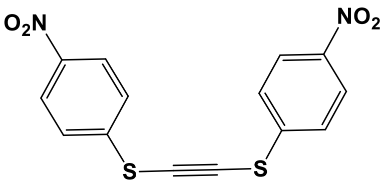

**Bi_nitro**

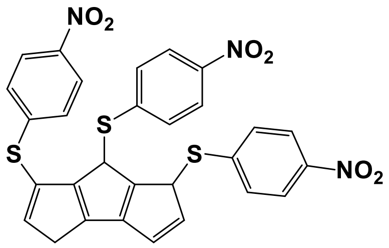

**Tri_nitro**

**Supplementary Figure 5 |** Structural of bi-ATP/NBT and tri-ATP/NBT

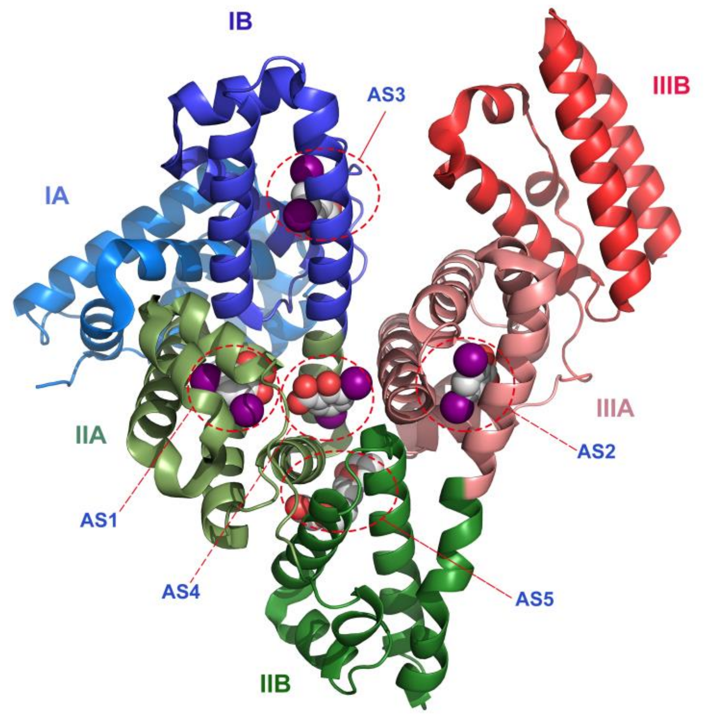


**Supplementary Figure 6 |** Structure and proposed active binding sites (AS) of bovine and human serum albumin, the cartoon representation (PDB ID: 4JK4) is generated by PyMOL™ v1.7 sofeware. AS1 is located in subdomain IIA, AS2 in subdomain IIIA, AS3 in domains IB, AS4 inside the cavity between IIA and IIIA, close to the first AS1.

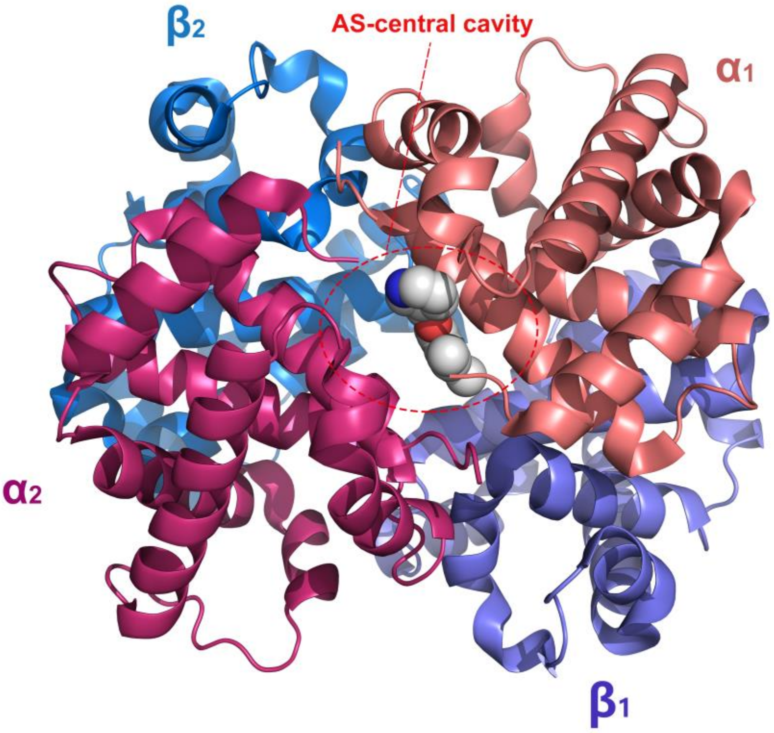


**Supplementary Figure 7 |** Structure and central cavity binding site of bovine hemoglobin, the cartoon representation (PDB ID: 3R5I) is generated by PyMOL™ v1.7 sofeware. A surface pocket is located at the central cavity of the α1 α2 subunits。

**Reference:**


1. Lacerda, S. H. et al. Interaction of gold nanoparticles with common human blood proteins. *ACS Nano* **4**, 365-379 (2010).
2. Li, N., Zeng, S., He, L., Zhong, W. Probing nanoparticle-protein interaction by capillary electrophoresis. *Anal. Chem.* **82**, 7460-7466 (**2010)**.
3. Gebauer, J. S. et al. Impact of the nanoparticle-protein corona on colloidal stability and protein structure. *Langmuir* **28**, 9673-9679 (2012**)**.
4. Chu, Y., Lees, W. J., Stassinopoulos, A. & Walsh, C. T. Using affinity capillary electrophoresis to determine binding stoichiometries of protein-ligand interactions. *Biochemistry* **33**, 10616-10621 (1994).
5. Galbusera, C., Thachuk, M., Lorenzi, E. D. & Chen. D. D. Y. Affinity capillary electrophoresis using a low-concentration additive with the consideration of relative mobilities. *Anal. Chem.* **74**, 1903-1914 (2002).
